# Cross-sectional survey in Central African Republic finds mortality 4-times higher than UN statistics: how can we not know the Central African Republic is in such an acute humanitarian crisis?

**DOI:** 10.1186/s13031-023-00514-z

**Published:** 2023-04-18

**Authors:** Karume Baderha Augustin Gang, Jennifer O’Keeffe, Anonymous, Les Roberts

**Affiliations:** 1 Rebuild Hope for Africa & Doctoral Candidate, Université Evangélique en Afrique (UEA), Bukavu, Democratic Republic of the Congo; 2 Johns Hopkins Bloomberg School of Public Health, Baltimore, MD USA; 3 Bocaranga, Central African Republic; 4 Columbia University Mailman School of Public Health, New York, NY USA

**Keywords:** Mortality, Central African Republic, UN Statistics, Humanitarian crisis, Violence

## Abstract

**Background:**

CAR is one of the poorest countries in the world. While UN statistics suggest that there is no health emergency in the country, two recently published mortality surveys contradict this. Moreover, recent accusations of massive scale human rights abuses by mercenaries suggested the need for a nationwide mortality survey.

**Methods:**

Two stage cluster surveys were conducted in two different strata: one in the roughly half of the country within the Government’s control, and one in the areas mostly outside of the Government’s control. We randomly selected 40 clusters of 10 households in each stratum. The survey included questions on vital events with open-ended questions about health and household challenges at the beginning and end of each interview.

**Results:**

70 of 80 selected clusters were successfully visited. We interviewed 699 households, containing 5070 people. 11 households (1.6%) refused to be interviewed and approximately 18.3% of households were absent at the time of visitation, mainly in the safer Government controlled areas. Interviewed households had a birth rate of 42.6 /1000 / year (95%CI 35.4–59.7) and a crude mortality rate (CMR) of 1.57 /10,000/day (95%CI: 1.36–1.78). The birth rate was lower and the death rate markedly higher in the strata outside of Government control. Families described malaria or fever, and diarrhea as the primary reported causes of death with violence accounting for 6% of all deaths.

**Conclusions:**

CAR is experiencing a severe health emergency, with the highest measured nationwide mortality in the world to our knowledge. UN published death rate estimates appear to be less than one fourth of reality. There is a desperate need for food aid in the form of general distributions in CAR, along with the accompanying work programs, seed and tool distributions needed to restart local economies. This is of particular importance in rural areas outside of the Government control. While some humanitarian actors are doing their best to respond, the crisis level mortality rate suggests that the needs in CAR are being largely unmet.

## Background

The Central African Republic has a gross national income of $425 per person, a life expectancy of 54, and is ranked fourth from last on the Human Development Index [1, 2]. For the past two decades, much of the country has been in the control of anti-government rebels. Seleka rebels overtook the capital in 2013–14, with major flare-ups occurring again in 2017 and 2020 [3]. Conditions have reportedly changed since 2020 as mercenaries from the Wagner group have expanded their activities in rebel held parts of the country. Wagner’s presence was first documented in 2018. They have been accused of widespread killings, public executions, torture, and rape, as strategies of demoralization and disruption [4, 5].

Following the publishing of 1992 Centers for Disease Control guidelines for public health emergencies, an acute health crisis has been widely accepted as occurring when the baseline mortality has either doubled or exceeded the rate of 1/10,000/day [6, 7]. While psychological stress, disruption of life quality, food insecurity, and risk of outbreaks can all trigger a humanitarian response, the doubling of mortality is an exceptionally apolitical and effective measure for doing so. According to the official UN Statistics, CAR has a relatively low crude mortality rate (12/1000/yr. or 0.33/10,000/day), similar to those reported for Lesotho and Nigeria. These rates have reportedly been declining steadily since 2001 despite the political turmoil and violence [8].

Two surveys published over the last two years have suggested that large areas of the country were experiencing mortality above the emergency threshold [9, 10]. In 2022, UNICEF announced that food insecurity and malnutrition were widespread and threatened the death of tens of thousands of children [11]. They only reached half of their new funding goals [12]. Because of the discordance of recent mortality measures and the official mortality rate, accusations of recent widespread killings and human rights abuses, and the multifaceted crises that have arisen, a careful, nationwide measure of mortality in CAR was needed. Thus, this survey attempts to: 1) measure the nation’s CMR, 2) better understand the discordance between mortality measures.

## Methods

To increase the precision of our mortality estimate, the national population was divided into two strata. The first, included the 52.5% of the population living in areas served and controlled by the national Government (GC). These included: the city of Bangui, and Ombella-M'Poko, Lobaye, Mambéré-Kadeï, Nana-Mambéré, Sangha-Mbaéré, Kémo Prefectures. The second strata included the remaining prefectures which were assessed by the national team members to be less stable, less supported by Governmental services, and partially out of the Government’s control (Ouham-Pende, Ouham, Ouaka, Nana Gabriza, Baningui Bangoran, Vakaga, Basse-Kotto, Haute-Kotto, Haut-Mbomou, and Mbomou Prefectures, estimated to be 47.5% of the population), hereafter referred to as non-GC. This assessment of government control aligned with the assessment of a July 2021 map drawn by Political Geography Now [13].

The survey sample size estimate was based on assumptions that: the baseline mortality was 1.0/1000/mo. (~ 0.33/10,000/day) [8], each strata sample should be able to detect a 50% elevation of that rate, the average household would be 5.0 people [9, 10], the design effect (DEFF) of the cluster sampling would be 1.5, and there would be a 10-month recall period. The 10-month recall allowed the use of the start of 2022 as a date of reference in interviews. This suggested a sample of 1059 people in each stratum (WinPepi 11.65). We doubled the required sample to allow for adequate precision in the event that security or logistic constraints prevented us from visiting all clusters. For the final sample, we selected 40 clusters of 10 households in each stratum (2000 people per strata).

The survey was conducted in each stratum as a two-stage cluster design [14]. In stage 1, we assigned clusters to prefectures systematically, probability proportional to size. Prefecture population estimates were based on the 2019 CAR official population estimates by the Institute Centrafricain des Statistiques et des Etudes Economique et Sociale (ICASEES) [15]. A satellite image-based database was made available to the team that included the latitude and longitude of each building in the country and assigned it a number. (Central African Republic Ecopia Building Footprint layer, ©2019 Digital Globe, Inc.) For stage 2, we randomly selected an individual building from the image database for each cluster starting point. If no household existed at the assigned spot, the study team visited the nearest location with at least 10 households. If we estimated that we could not travel to and from a cluster within 10 h by motorcycle or car from where the team spent the night, or if insecurity was assessed to be extremely high, the cluster was skipped, or the nearest reachable village used as a substitute.

We sought approval from Bangui and local administrative officials before visiting households in the clusters. We asked village or neighbourhood leaders if there had been any general population food distributions, and if there had been fighting between armed groups within 10 km of the village in 2022. We used GPS devices, phones, or in the riskiest areas, hand-drawn maps to identify the starting point of a cluster. Interviewers approached the 10 households nearest to the starting point. At each household, interviewers sought informed consent, by describing the purpose of the study, indicating there would be no direct rewards or benefits to participants and specifying that participation was completely voluntary. Interviewers sought verbal consent due to the low levels of literacy in most of CAR and the potential risk posed to interviewees if we sought written consent. If the interviewee agreed to participate, the interviewer signed a consent line on the data form.

To be eligible for the study, the consenting individual needed to be 18 years of age and a resident in the visited household. A household was defined as a group of people who slept under the same roof or ate their meals together. Interviewers confirmed with respondents that household members had been present most of the last 30 nights. Those members who did not meet the criteria were excluded. No identifying information was collected on the data forms beyond household demographics. If a household refused to be interviewed, or was absent at the time of the visit, it was passed over and the next nearest household to the starting point was visited. We replaced empty households because past surveys found that rural villages had more empty households than urban and corrective weighting of clusters would have introduced bias. Because of logistical barriers, clusters were not revisited after the initial visit. In some areas, interviewers used local moto-taxis to access clusters as local drivers were less likely be attacked by armed groups. In such visits, no team leader was present and proper absentee data was not recorded, though refusal data was.

The questionnaire consisted of 15 questions beginning with an open-ended question about the health challenges facing the household. This was followed by questions adopted from Medecins Sans Frontieres (MSF)’s mortality survey protocol on household demographics, pregnancies, births, deaths, household migration, and duration of residence. Finally, households were asked if they had received food in distributions of any kind in 2022. The survey concluded with an open-ended question asking if there was anything else they wanted to share. The inclusion of the open-ended questions provided households with an opportunity to raise concerns that may not have been anticipated in the pre-developed questionnaire. Interviews took approximately 15 min. We trained interviewers on the survey methods, the informed consent process, and all segments of the questionnaire, including household responses to open ended questions. The questionnaire was translated and back translated from French to Sango. Interviewers conducted the interviews in whatever language provided the best communication with respondents.

Interviewers recorded answers on paper forms that were quality checked by team leaders in the field. Data was entered using Kobo Toolbox [16] and 10% of entries were independently checked for quality control. We conducted all analysis in R version 4.2.1 with RStudio version 2022.07.2 + 576. For quantitative results, we applied survey weights to the sample using population estimates of the strata. We calculated demographic, pregnancy outcome, mortality and reported cause of death estimates, with corresponding 95% confidence intervals, and association testing. We calculated design effects for births and death results. We searched responses to the open-ended questions for key words and themes during analysis.

The study was funded by an anonymous donor who had no communication with the team about study design, fieldwork undertaken, or results. IRB approval was obtained from The Comite National D’ethique de la Sante of the DRC Ministry of Health, CNES 001/DPSK/195PM/2022.

## Results

We collected data from 70 of the 80 selected clusters, covering 14 of 17 prefectures. Seven clusters were missed because of security concerns (n = 6 in non-GC areas) and three because of logistical concerns (n = 2 in GC areas). Of the sampled clusters, 37 were in GC areas and 33 in non-GC areas. We substituted the nearest accessible village in eight clusters, five in GC areas, all due to logistic barriers and three in non-GC areas, all due to security concerns. The median distance of the substitutes was 16 km (range 15–45 km) to the selected cluster in GC areas and 10 km (range 9–25 km) in the non-GC areas. Active combat between armed groups was reported in 22 of 33 (66.7%) non-GC clusters and 0 of the GC clusters. Of the 22 non-GC clusters with active combat, 19 local chiefs reported involvement with “Russian” groups.

We visited 856 households, of which 699 (81.6%) were included in the study. Of 352 households visited in the non-GC areas with a team leader, we recorded 22 (6.3%) absent and 0 refusals. Of 504 households visited in the GC areas with a team leader, we recorded 124 (24.6%) absent and 11 (2.2%) refusals. The median non-GC household size was 6 people (IQR 4–7; mean 6.0; 95%CI 5.8–6.3). The median GC household size was 7 people (IQR 5–9; mean 7.4; 95%CI 7.0–7.7). Interviewed households included 5,070 individuals present at some point in 2022, 2,168 in the non-GC areas and 2,902 in the GC areas.

### Study population

**Fig. 1:**
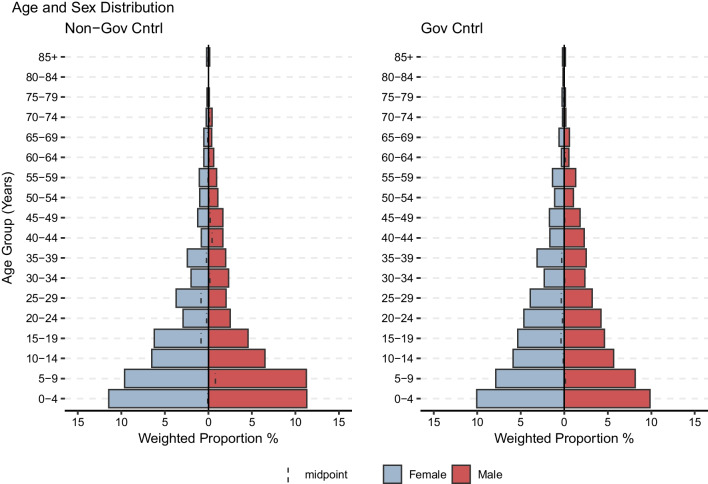
Age and sex breakdown of survey strata

**Fig. 2:**
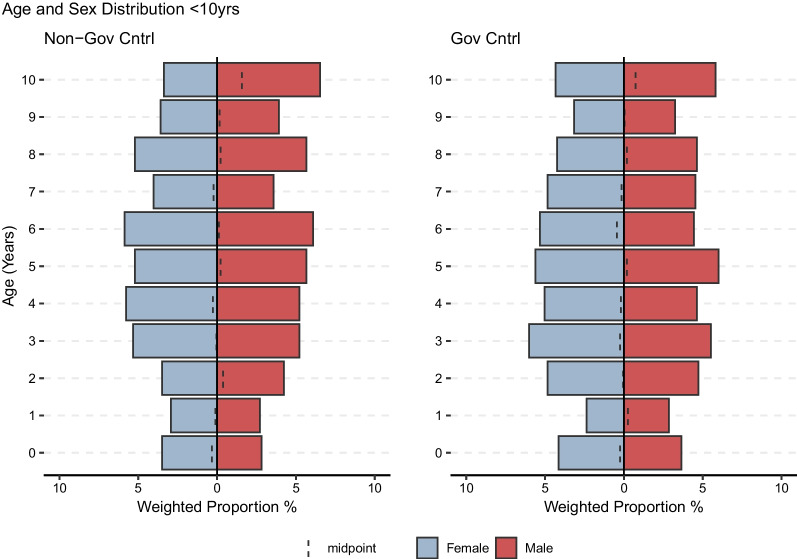
Age and sex breakdown among those < 10 years of age

The median age of the study population was 15 (IQR: 6–30). See Fig. 1 below. The median age was lower in the non-GC areas than in the GC areas (13 (IQR: 6–28) vs. 17 (IQR: 7–32), *p*-value < 0.001). Females made up 50.8% (95%CI: 49.4–52.2) of the total population, 50.5% (95%CI: 48.4–52.6) in the non-GC areas and 51.0% (95%CI: 49.1–52.8) in the GC areas (*p*-value 0.73). Children < 5 years old comprised 17.1% (95%CI 16.1–18.2) of the total population, 18.0% (95%CI 16.4–19.7) in the non-GC areas, and 16.3% (95%CI: 15.0–17.7) in the GC areas (*p*-value 0.13). Figure 2 shows the age breakdown for children 10 years of age and younger.

### Birth rates and pregnancy

There were 157 reported pregnancies in the non-GC areas and171 in the GC areas in 2022. The crude birth rate was 39.0 (95%CI: 28.6–49.3) per 1000 per year in the non-GC areas and 46.1 (95%CI: 36.2–55.9) in the GC areas. The overall birth rate was 42.6 (95%CI 35.4–59.7). Of the population surveyed, 4.0% (n = 79) of females were currently pregnant in the non-GC areas and 2.4% (n = 64) in the GC areas. Of the reported pregnancy outcomes, 25.5% (95%CI: 19.7–32.4, DEFF: 1.05) resulted in a pregnancy loss across the survey (32.1% (95%CI: 22.5–43.4) in the non-GC areas and 19.6% (95%CI: 13.1–28.4) in the GC areas, *p*-value: 0.06). The remaining pregnancies resulted in a birth (Table 1).

**Table 1:** Pregnancy Outcomes (Number and Weighted Proportions)

	Overall N = 185			Non-Government N = 78			Government N = 107			*p*-value ^a^
	n	%	95% CI	n	%	95% CI	n	%	95% CI	
Birth	139	74.5	67.6–80.3	53	67.9	56.6–77.5	86	80.4	71.6–86.9	0.06
Loss	46	25.5	19.7–32.4	25	32.1	22.5–43.4	21	19.6	13.1–28.4	0.06

^a^Chi-squared test with rao and scott second order correction

### Mortality rates and cause of death

There were 115 deaths in the non-GC areas and 105 deaths in the GC areas (Table 2). We estimated the crude mortality rate (CMR) at 1.85 (95%CI: 1.51–2.19) in the non-GC areas and 1.31 (95%CI: 1.06–1.56) in the GC areas. The overall CMR was 1.57 (95%CI: 1.36–1.78, DEFF: 1.02). In the non-GC areas, the under-five mortality rate (U5MR) was 2.62 (95%CI: 1.63–3.61) compared to 1.08 (95%CI: 0.49–1.66) in the GC areas. The overall U5MR was 1.86 (95%CI: 1.28–2.43, DEFF: 1.04). The male-specific mortality rate was 2.22 (95%CI: 1.69–2.75) in the non-GC areas and 1.43 (95%CI 1.05–1.81) in the GC areas. The distribution of deaths by sex was similar with male deaths comprising 59.1% (95%CI: 49.8–67.8) in the non-GC areas and 54.4% (95%CI: 44.6–63.8) in the GC areas (*p*-value 0.48).

**Table 2:** Mortality rates per 10,000 per day (Number and weighted rate estimates)

	Overall			Non-Government			Government		
	n	Estimate	95% CI	n	Estimate	95% CI	n	Estimate	95% CI
CMR	220	1.57	1.36–1.78	115	1.85	1.51–2.19	105	1.31	1.06–1.56
U5MR	40	1.86	1.28–2.43	27	2.61	1.63–3.60	13	1.08	0.49–1.66
Male	124	1.81	1.49–2.13	68	2.22	1.69–2.74	56	1.43	1.05–1.80
Female	94	1.32	1.05–1.58	47	1.49	1.07–1.92	47	1.16	0.83–1.49

The leading reported cause of death overall in both strata was malaria/fever, comprising 18.6% (95%CI 14.0–24.4) of all deaths: 18.3% (95%CI: 12.2–26.5) in non-GC areas and 19.0% (95%CI: 12.6–27.8) in GC areas. In the non-GC areas, the second and third leading reported causes of death were diarrhea/vomiting (9.6% (95%CI: 5.3–16.5) and hernia (8.7% (95%CI: 4.7–15.5). In the GC areas general pain/sickness was the second leading reported cause of death (9.5% (95%CI: 5.2–16.9) with diarrhea/vomiting and non-communicable diseases tied for third (7.6% (95%CI: 3.8–14.6). Of all deaths, 47.5% (95%CI: 40.8–54.3) died at home, 44.8% (95%CI 38.1–51.6) died at a health facility, 2.3% (95%CI 1.0–5.5) died en route to a health facility, and 5.4% (95%CI: 3.1–9.3) died in another location. These proportions were similar for non-GC and GC areas in Table 3 (*p*-value: 0.14). Of deaths, 50.0% (95%CI: 43.3–56.7) were reported to have sought care, without significant differences between the strata (*p*-value: 0.59).

**Table 3:** Reported cause of death (Number and weighted proportions)

Reported cause of death	Overall N = 220			Non-Government N = 115			Government N = 105		
	n	%	95% CI	n	%	95% CI	n	%	95% CI
Malaria/fever	41	18.6	14.0–24.4	19	18.3	12.2–26.5	18	19.0	12.6–27.8
Diarrhea/vomiting	19	8.7	5.6–13.3	11	9.6	5.3–16.5	8	7.6	3.8–14.6
General pain/sickness	14	6.1	3.6–10.1	4	3.5	1.3–9.0	10	9.5	5.2–16.9
Non-communicable diseases ^a^	14	6.2	3.7–10.3	6	5.2	2.3–11.2	8	7.6	3.8–14.6
Other stomach/GI issues	14	6.5	3.9–10.7	9	7.8	4.1–14.5	5	4.8	2.0–11.0
Violence	14	6.4	3.8–10.6	8	7.0	3.5–13.4	6	5.7	2.6–12.2
Communicable diseases ^b^	12	5.3	3.0–9.1	4	3.5	1.3–9.0	8	6.7	3.8–14.6
Hernia	10	5.0	2.7–9.0	10	8.7	4.7–15.5	0	0	–
Inflammation/swelling	10	4.6	2.5–8.4	6	5.2	2.3–11.2	4	3.8	1.4–9.8
Accident	8	3.6	1.8–7.1	4	3.5	1.3–9.0	4	3.8	1.4–9.8
Anemia	8	3.8	1.9–7.4	6	5.2	2.3–11.2	2	1.9	0.5–7.4
Old age	8	3.6	1.8–7.1	4	3.5	1.3–9.0	4	3.8	1.4–9.8
Unknown	8	3.3	1.6–6.4	0	0	-	8	7.6	3.8–14.6
Respiratory/lung	7	3.2	1.5–6.6	4	3.5	1.3–9.0	3	2.9	0.9–8.6
Cardiac	5	2.0	0.8–4.8	0	0	–	5	4.8	2.0–11.0
Infection	4	1.9	0.7–5.0	3	2.6	0.8–7.9	1	1.0	0.1–6.6
Meningitis	4	1.9	0.7–5.0	3	2.6	0.8–7.9	1	1.0	0.1–6.6
Other^c^	4	1.8	0.7–4.8	2	1.7	0.4–6.8	2	1.9	0.5–7.4
Paralysis	4	1.7	0.6–4.5	1	0.9	0.1–6.0	3	2.9	0.9–8.6
Convulsions	3	1.5	0.5–4.5	3	2.6	0.8–7.9	0	0	–
Snake bite	3	1.4	0.4–4.3	2	1.7	0.4–6.8	1	1.0	0.1–6.6
Malnutrition	2	1.0	0.2–3.6	2	1.7	0.4–6.8	0	0.0	–
Neonatal	2	0.9	0.2–3.6	1	0.9	0.1–6.0	1	1.0	0.1–6.6
Pregnancy/birth complication	2	0.9	0.2–3.6	1	0.9	0.1–6.0	1	1.0	0.1–6.6

^a^Includes: Hypertension (n = 7), diabetes (n = 3), diabetes + hypertension (n = 2), prostate disease (n = 1), and cancer (n = 1)

^b^Includes: Flu (n = 6), yellow fever (n = 2), typhoid (n = 2), measles (n = 1), and sleeping sickness (n = 1)

^c^Includes: Blood problems (n = 1), skin rash (n = 1), spleen problems (n = 1), and suicide (n = 1)

### Household challenges

Households reported a reduction in the number of meals eaten per day before versus after the crisis for adults (2.68 vs. 1.18, *p*-value: < 0.001) and children (2.76 vs. 1.28, *p*-value: < 0.001). Of all households, 82.3% (95%CI: 79.4–84.9) reported ≤ 1 meals a day for adults and 72.6% (95%CI: 69.4–75.6) reported ≤ 1 meals a day for children. When households were asked if they would like to share any additional information, the most mentioned subjects were needs related to health (26.9%), food (25.2%), and lack of money (11.3%).

## Discussion

The mortality measured in this nationwide survey is over four times higher than the United Nations’ estimates, more than twice reported for any other country in the world, and well above the emergency threshold. This compels us to raise several questions about this measure: is the United Nations (UN) estimate off by four-fold, is this survey measurement incorrect, and is the extremely high mortality caused by recent events?

Keuhne et al. described a large body of work suggesting that the UN mortality estimate is implausibly low [17]. Table 4 below summarizes the key measures of those sources and others.

**Table 4:** various survey estimates of death rates (per 10,000/day) and fraction of population < 5

When	Who/what	Where	CMR	< 5MR	% pop. < 5, (birth rate)
2021	UN modeled estimates	Nation [8]	.33	~ .6	3.5% est
2022	RHA survey	Nation (this survey)	1.57 (1.36–1.78)	1.86 (1.28–2.43)	17.1% (4.3%)
2020	MSF survey	Ouaka [9]	1.33 (1.09–1.61)	1.87 (1.37–2.54)	22.8% (5.9%)
2019	SMART survey. MOH, UNICEF, WFP	Nation [18]	0.84	1.12	21.7%
2019	SMART survey. MSF OCBA	Alindao & Mingala. Prefecture Basse Kotto. [17]	1.93 (1.55–2.40)	3.47 (2.54–4.70)	21.1%
2018	IRC/Columbia Univ. survey	Ouham Pende [10]	1.3	1.7	22% (5.4%)
2018	SMART survey. AHA	Commune Mobaye, Prefecture Basse Kotto [19]	0.37 (0.21–0.65)	0.75 (0.35–1.60)	?
2018	SMART survey. AHA	Lagandi, Commune Mbelima, Sous-prefecture Mobaya, Prefecture Base Kotto. [19]	0.55 (0.32–0.96)	1.52 (0.79–2.92)	?
2018	SMART survey. AHA	Ouambe. Sous-prefecture Zangba.Prefecture Basse Kotto. [19]	–	2.3 (1.7–3.5)	?
2012	MSF mortality survey	Boguila, Northern Ouham Prefecture. [19]	0.84	1.77	23%
2010	MSF surveillance	Boda, Boganangone, Boganda, Gadzi w/in Lobaye^see^ [17]	1.0	2.0	19.9% (6.2%)
2010	Berkeley Univ	Bangui, Lobaye, Ombella M’Poko, Ouham, Ouham-Pende Prefectures [19]	1.6 (1.5–1.7)		
2009	UNICEF / Columbia Univ	Nation [20]	2.0 (1.7–2.3)	2.6 (1.6–3.5)	17.7%

Table 4 suggests that this UN underestimation of mortality has been going on for years, or  perhaps decades. UN mortality estimates are generally based upon standardized DHS or MICS surveys, which are primarily conducted in non-conflict settings, and typically cost over a million dollars each [21]. Studies in the region have shown that such surveys which attempt to capture multi-year maternal birth histories and estimate < 5 mortality at the same time, grossly under-record mortality [22, 23]. Some have criticized the UN model-based estimates as founded on flawed survey techniques [24] which get used for further model inputs, exacerbating the gaps between projections and reality [25]. Many assumptions used in modelled estimates, including that birth and deaths rates change steadily over 5 year periods, are inappropriate in conflict settings. The nature of conflict settings is inevitably volatile, suggesting that only very short-term and limited assumptions may be suitable. With pressure to show progress on the Sustainable Development Goals, both the UN and the Government are in a challenging position. Both are invested in the conflict; the Government directly over control of its own sovereign territory and the UN in support of the Government, and through its Peacekeeping mission. It may be that host governments and the allied United Nations that support them are not capable of producing reliable mortality estimates during conflicts.

The 2010 MICS survey done jointly with UNICEF and the Bangui Government, which produced the last official UN CMR national estimates we have seen, sampled only areas controlled by the government, and only from villages reachable by vehicle. In rural areas of our survey, teams with motorcycles were 2.6 times more likely to reach the sampled location than a vehicle. Further undermining the 2010 MICS survey’s credibility are reports that in some places interviewers were accompanied by armed military escort, an act which would refute any claim of neutrality in the findings. (LR personal communication with MICS 2010 interviewers who later worked for him on Ref. 26). Given the significance that measures of mortality play in identifying humanitarian emergencies, this survey, conducted by a small, regional NGO with a total budget of $50,000, raises the question: are there better ways to bring global health crises to the world’s attention?

There are several major limitations to our survey. We missed 10 of 80 clusters selected, three in the GC areas, seven in the non-GC. Most of these were missed due to insecurity, meaning the most at-risk populations were not included. Secondly, a similar survey in DRC documented survivor bias from deaths in households that had dissolved, which we would have missed [26]. A large portion of households in the GC areas were absent at the time of interview. The cause of death data was based on what families reported. While half of deaths (111/220, 50.5%)) sought care for their final illness, the precision of the diagnoses are not known. Our second stage sampling by satellite images likely resulted in under-sampling of people in multi-story apartments. As there are very few multi-story residential buildings outside of downtown Bangui, this bias is unlikely to be substantial. In sum, if this mortality survey is incorrect, we believe our measure is more likely to be an under-estimate rather than an over-estimate of the mortality rates in CAR.

In answer to the last question posed above: the evidence suggests things may indeed have gotten worse over the last two years. When speaking of the present crisis, respondents referred to one of two things that developed in 2020. In the GC areas, many associate the present crisis with the arrival of COVID and its associated disruptions as well as the efforts of  the Wagner Group to regain national territory after a decades-long hold by rebel groups. [4, 5]. There have been widespread human rights abuses by the Wagner group in these efforts, including public executions, massacres, and rape as a weapon of war, mainly in areas thought to be rebel strongholds [4, 27]. This has had the effect of driving armed combatants to more frequent and severe confrontations with civilians in order to survive. Dozens of households in the Non-GC areas described not being able to travel to visit their fields, hunt, fish or forage for survival because combatants would attack or kill them.

It is difficult to articulate the suffering associated with this elevated mortality. Many indicators aside from the death rate confirmed or validated the severity of the crisis. We ended each interview with an open-ended inquiry asking if respondents had additional information to share. Most people expressed that they were desperate for assistance, asking for health care (26.9%) and food (25.2%). In this region, it is unusual to have more deaths reported in households than births as  we measured in this survey. A disturbing 82.3% of households reported adults ate ≤ 1 meals per day at the time of interview. Similarly, 72.6% of children, who have greater nutritional needs and are at higher risk for malnutrition and its associated morbidities, ate ≤ 1 meals per day at the time of interview. The demographic profile of children under 10 (see Fig. 2), shows an acute dearth of children < 3 in a population with low contraceptive use, implying either a recent elevation of infant mortality, a decrease in birth rates due to extreme stress, or both. Corroborating the likelihood of a reduced birth rate, 25.5% of known pregnancies resulted in a loss. This is higher than the fraction recorded in neighboring Eastern DRC in 2002 during a period of extreme conflict [28]. All of these factors, and their relative severity in the non-GC areas, suggests that the efforts of the Wagner mercenaries at least contributed to increased difficulties of survival over the past two years.

Humanitarian actors have been raising the alarm of the severity of the situation in CAR for over a decade [29]. The UN has been warning of extreme food insecurity for two years [12]. To the credit of certain agencies, households report food distributions in some of the most critical areas. Five GC clusters and 12 Non-GC reported distributions from World Food Program (WFP),  Action Contre la Faim (ACF), and others in 2022. Our findings show that despite these efforts, aid to the population of CAR is woefully insufficient. With a budget > 233 million USD in 2022 for a population of approximately five million from the US Government alone [30], one has to ponder if the humanitarian community can do better? In particular, are we ignoring one of the world’s worst humanitarian crisis?

### Recommendations

This study cannot distinguish the relative importance of decades of ongoing conflict, extreme poverty, the economic disruptions since 2020, or the widespread disruption efforts of the Wagner Group in causing the extreme mortality observed in CAR. Thus, the following measures seem paramount, with little chance of regret.

1. Given the United Nations’ apparent gross under-estimate of deaths in CAR, there is a need for an independent, experienced group with no direct association with the UN or warring parties to confirm this mortality finding.
2. There is a desperate need for food aid in the form of general distributions in CAR, along with the accompanying work programs, seed and tool distributions needed to restart local economies. This is of particular importance in rural areas outside of the Government control. WFP and several NGO’s have been doing this well. Their efforts were greatly appreciated by the 18% households that received aid in 2022. Despite this, the crisis level mortality rate suggests that the needs in CAR are being largely unmet.

## Data Availability

The anonymized datasets used and/or analysed during the current study are available from the corresponding author Karume Baderha Augustin Gang on reasonable request, at gangkarume@yahoo.fr.

## Abbreviations

ACF: Action contra la Faim
CAR: Central African Republic
CMR: Crude mortality rate
DHS: Demographic health surveys
GC: Government controlled areas
MICS: Multiple indicator cluster survey
MOH: Ministry of Health
MSF: Médecins sans frontières
NGO’s: Non-governmental organizations
Non-GC: Areas not controlled by the government to some extent
RHA: Rebuild Hope for Africa
SMART: Standardized monitoring and assessment of relief and transitions
UN: United Nations
UNICEF: United Nations International Children’s Emergency Fund
US: United States
WFP: World food program
